# Immunological Properties of Corneal Epithelial-Like Cells Derived from Human Embryonic Stem Cells

**DOI:** 10.1371/journal.pone.0150731

**Published:** 2016-03-15

**Authors:** Zhenyu Wang, Qingjun Zhou, Haoyun Duan, Yao Wang, Muchen Dong, Weiyun Shi

**Affiliations:** 1 Qingdao University, Qingdao, Shandong, China; 2 Shandong Provincial Key Laboratory of Ophthalmology, Shandong Eye Institute, Shandong Academy of Medical Sciences, Qingdao, Shandong, China; Rutgers University -New Jersey Medical School, UNITED STATES

## Abstract

Transplantation of ex vivo expanded corneal limbal stem cells (LSCs) has been the main treatment for limbal stem cell deficiency, although the shortage of donor corneal tissues remains a major concern for its wide application. Due to the development of tissue engineering, embryonic stem cells (ESCs)-derived corneal epithelial-like cells (ESC-CECs) become a new direction for this issue. However, the immunogenicity of ESC-CECs is a critical matter to be solved. In the present study, we explored the immunological properties of ESC-CECs, which were differentiated from ESCs. The results showed that ESC-CECs had a similar character and function with LSCs both in vitro and in vivo. In ESC-CECs, a large number of genes related with immune response were down-regulated. The expressions of MHC-I, MHC-II, and co-stimulatory molecules were low, but the expression of HLA-G was high. The ESC-CECs were less responsible for T cell proliferation and NK cell lysis in vitro, and there was less immune cell infiltration after transplantation in vivo compared with LSCs. Moreover, the immunological properties were not affected by interferon-γ. All these results indicated a low immunogenicity of ESC-CECs, and they can be promising in clinical use.

## Introduction

The limbal stem cells (LSCs) located at the palisades of Vogt serve as the source of renewal and repair of the corneal epithelium, and a barrier to prevent the aggression of neighbouring conjunctival epithelium [1–4]. Many conditions, such as thermal or chemical burn, Stevens-Johnsons syndrome, autoimmune diseases, and contact lens wearing, can lead to limbal stem cell deficiency (LSCD), resulting in conjunctivalization of the corneal surface, recurrent and persistent epithelial defects, chronic inflammation, scarring and ulcerations of the cornea [5–8]. Patients with LSCD suffer not only visual loss but also continuous pain.

Currently, the main treatment for LSCD is surgical intervention. Transplantation of corneal limbal tissue, ex vivo expanded LSCs, and oral mucosal epithelial cells was reported in succession for the ocular surface reconstruction [9–11]. Although good outcomes were achieved in many cases, the disadvantages of deficient donor tissue and immune rejection limit their wide application. The corneal epithelial-like cells derived from pluripotent stem cells, especially embryonic stem cells (ESCs), provides an ideal source of donor cells for the LSCD treatment. Many studies have reported a successful differentiation of ESCs into corneal epithelial-like cells (ESC-CECs) and the same phenotype with LSCs [12–15], and so as to our research team.

Before the ESC-CECs are applied clinically, it is critical to investigate the immunological properties of such cells. ESCs and their derivatives were believed to be lowly immunogenic because they expressed few major histocompatibility complex class I and II molecules (MHC-I and MHC-II) and co-stimulatory molecules [16, 17]. However, the rejection is a complicated reaction, and many other factors may influence the immunogenicity, e.g. the minor histocompatibility antigen, the long differentiation time, and the composition of culture medium [18–20]. Furthermore, when the donor cells are transplanted into the body, their immunogenicity may change greatly. The transplanted cells are more susceptible to immune cells in the inflammatory microenvironment [21, 22].

In this study, we explored the immunogenicity of corneal epithelial-like cells derived from human ESCs by comparing them with human corneal LSCs. No matter in vitro or in vivo, the ESC-CECs were found to be less immunogenic than the LSCs.

## Methods

### Ethics approval

The use and experimental protocol of human limbal tissue, human ESC line H1 and peripheral blood from healthy donors was approved by the Ethics Committee of the Shandong Eye Institute (No. SEIRB-2014-147C08). The research purpose and detailed experimental protocol was informed to the donor of healthy peripheral blood and the next kin of human limbal tissue donor. They agreed and signed written informed consent for the use of this sample in research.

The animal experiment was approved by the Ethics Committee of the Shandong Eye Institute (No. SEIRB-2014-168A11). All operations were performed following the Association for Research in Vision and Ophthalmology (ARVO) guidelines concerning the use of animals in ophthalmic and vision research.

### Cell culture and differentiation

The human LSCs were obtained from limbal tissue of fresh donor eyes as previously reported [23]. Briefly, the limbus was digested by 2.4 U/ml Dispase (Sigma, St. Louis, MO) at 4°C overnight, and the epithelium was scraped with an epithelial scraper. After repeated pipetting with 0.05% trypsin, the single cell suspension was seeded on 3T3 feed layer in limbal stem cell medium, and LSCs were obtained after 7 days. The limbal stem cell medium was composed of DMEM/F12 (3:1), 10% fetal bovine serum, antibiotics, hydrocortisone, cholera toxin, transferrin, L-glutamine, and recombinant human EGF [24].

The human ESC line H1 was given by China Key Lab of Ophthalmology of Chinese People's Liberation Army [25]. It was cultured and passaged in mTeSR™1 defined, feeder-free maintenance medium (Stemcell technologies, Vancouver, BC). The differentiation was induced in an unconditioned medium with 1 μM retinoic acid (RA, Sigma, St. Louis, MO), 10 μM TGF-β inhibitor SB505124 (Sigma, St. Louis, MO), and 10 μM IWP-2 (Merck, Darmstadt, Germany) for 7 days. Then the cells were seeded at 1.5×10^4^/cm^2^ on gelatin-coated plates in defined keratinocyte serum-free medium with 5% fetal bovine serum and grew until 70% fusion to obtain ESC-CECs.

To simulate the inflammatory influence on the immunogenicity, part of ESC-CECs and LSCs were treated with 25 ng/ml recombinant human interferon-γ (INF-γ, Millipore, Temecula, CA) for another 2 days. For transplantation, ESC-CECs and LSCs were digested and seeded on denuded amniotic membrane (dAM) with Transwell system in limbal stem cell medium [24]. After 7 days, these cells formed a stratified cell layer and were transplanted onto the ocular surface.

### Whole genome microarray study and analysis

The total RNAs of ESC-CECs and human LSCs were extracted by TRIzol after the cells were harvested. The Whole Human Genome Oligo Microarray (4x44K, Agilent Technologies, Santa Clara, CA) analysis was performed by Kangchen Biotechnology (Shanghai, China). Briefly, RNA quantity and quality were measured by NanoDrop ND-1000. RNA integrity was assessed by standard denaturing agarose gel electrophoresis. Sample labeling and array hybridization were performed according to the Agilent One-Color Microarray-Based Gene Expression Analysis protocol. Agilent Feature Extraction software was used to analyze acquired array images. Quantile normalization and subsequent data processing were performed using the GeneSpring GX v12.1 software package. Differentially expressed (DE) genes between the two samples were identified through Fold Change filtering. Gene ontology (GO) analysis was performed in the standard enrichment computation method. The GO categories were derived from Gene Ontology and included three structured networks: biological process (BP), cellular component (CC), and molecular function (MF). The microarray data was available through the GEO database with accession number GSE70152.

### Immunofluorescence staining

For cell staining, cells on the coverslip were washed with PBS and fixed in 4% paraformaldehyde. For tissue staining, the whole cornea with limbus was carefully harvested and embedded in OCT compound. Eight-μm frozen sections were made and fixed in cold acetone, after which specimens were treated with 0.1% Triton X-100 for 30 min and blocked with 5% BSA for 1 h. Fluorescein conjugated or non-conjugated primary antibody at appropriate concentration was used for incubation at 4°C overnight. If necessary, specimens were then incubated with fluorescent second antibody at room temperature for 2 h. Finally, after stained with DAPI for 5 min, slides were mounted with mounting medium and observed with a fluorescence microscopy (Nikon, Tokyo, Japan). The primary antibodies used were as follows: anti-human p63, anti-human Ki67, anti-human ABCG2, anti-human CK3/12, anti-human ABCB5, anti-human SSEA4, anti-human Nanog, anti-human OCT4 (all from Abcam, Cambridge, MA), anti-rabbit CD11b, anti-rabbit CD161, anti-rabbit CD4, and anti-rabbit CD8 (all from Biolegend, San Diego, CA).

### Real-time RT-PCR

Total RNA was isolated from human ESCs, human LSCs, and ESC-CECs with RNeasy Mini Kit (Qiagen, Hilden, Germany) according to the manufacturer’s instruction. One microgram of RNA was converted into complementary DNA utilizing the first strand synthesis kit (Invitrogen, Carlsbad, CA). The real-time PCR analysis was then performed in an Applied Biosystems 7500 real-time PCR machine (Applied Biosystems, Foster City, CA) with the SYBR Green PCR reagent (Invitrogen, Carlsbad, CA). The cycling system was an initial denaturation cycle at 95°C for 10 sec, followed by 45 cycles at 95°C for 15 sec and 95°C for 1 min. GAPDH was used as an endogenous control gene, and the primer sequences used in the study are shown in Table 1.

**Table 1:** Primer sequences used in real-time RT-PCR.

	Forward sequence (5'-3')	Reverse sequence (5'-3')
Nanog	ACCTCAGCTACAAACAGGTGAAG	AGAGTAAAGGCTGGGGTAGGT
OCT4	GTACTCCTCGGTCCCTTTCC	CAAAAACCCTGGCACAAACT
p63	CCCTTACATCCAGCGTTTCG	TTGTCTGTGTGCTCTGGGAC
Integrin β1	GGGACACGCAAGAAAATCCG	TGCACGGGCAGTACTCATTT
Involucrin	GCCTTACTGTGAGTCTGGTTGA	GCAGTGGAGTTGGCTGTTTC

### Flow cytometry

After washed by PBS, ESC-CECs and LSCs were harvested and resuspended into 1×10^5^/ml. Then specimens were incubated in PE or FITC conjugated primary antibody at 4°C for 30 min and washed three time. Flow cytometric analysis was performed with an FACS Calibur flow cytometer (BD Biosciences, San Jose, CA), and the data was analyzed with FlowJo software (Tree Star, Stamford, CA). The primary antibodies included PE-conjugated anti-human HLA-ABC, FITC-conjugated anti-human HLA-ABC, FITC-conjugated anti-human HLA-DR, PE-conjugated anti-human CD86, FITC-conjugated anti-human CD80, PE-conjugated anti-human HLA-G, and the appropriate isotype-matched controls (all from eBioscience, San Diego, CA).

### Mixed T lymphocyte proliferation assay

Human peripheral blood mononuclear cells were obtained from peripheral blood of healthy donors by Lymphoprep (Stemcell technologies, Vancouver, BC) density gradient centrifugation. CD4+ T cells were purified from mononuclear cells by magnetic cell sorting through positive selection with anti-human CD4 antibody (Miltenyi Biotech, Sunnyvale, CA) according to the manufacturer’s instruction. Then 1×10^5^ T cells were added to each well of a flat-bottom 96-well plate as responder population. The ESC-CECs and LSCs were respectively treated with mitomycin C (Sigma, St. Louis, MO) at 37°C for 2 h before seeded into wells at a ratio of 0.1:1, 0.5:1, and 1:1, with T cells as stimulator population. The mitomycin C-treated allogeneic T cells at a ratio of 1:1 served as positive control. The mixed cells were cultured in 100 μl RPMI-1640 medium supplemented with 10% fetal bovine serum and 1% penicillin/streptomycin at 37°C for 5 d. At the end of co-culture, 10 μl WST-1 (Premix WST-1 Cell Proliferation Assay System, Takara Bio, Japan) was added to each well. After incubation at 37°C for 2 h, optical density (OD) was measured at 450 nm in the microplate spectrophotometer. The proliferation index was determined as follows: (OD value of mixed cells—OD value of ESC-CEC or LSC alone) / OD value of T cells alone.

### Natural killer (NK) cell cytotoxicity assay

NK cells were obtained from mononuclear cells by magnetic sorting with anti-human CD56 antibody. Appropriate numbers of NK cells were mixed with 3×10^3^ ESC-CECs or LSCs at various effector:target (E:T) cell ratios in a flat-bottom 96-well plate. After incubation at 37°C for 6 h, supernatants were collected to detect the lactate dehydrogenase (LDH) release with a CytoTox96 non-radioactive cytotoxicity assay kit (Promega, Madison, WI) according to the manufacturer’s instruction. Finally, the OD was measured at 490 nm in the microplate spectrophotometer. NK cells and ESC-CECs or LSCs alone served as effector cell and target cell spontaneous LDH release, respectively. ESC-CECs or LSCs lysed by lysis solution in the kit served as target cell maximal LDH release. The % lysis was determined as follows: (OD value of experimental LDH release—OD value of effector cells spontaneous LDH release—OD value of target cells spontaneous LDH release) / (OD value of target cells maximal LDH release—OD value of target cells spontaneous LDH release).

### LSCD model and cell transplantation

New Zealand white rabbits (male, weighing 1.5–2 kg, aged 2–4 months, Kangda Co., Shandong, China) were used for the establishment of LSCD models and cell transplantation. All rabbits were housed in standard condition and the health was observed daily. The surgery was performed under systemic and ocular surface anesthesia, and all efforts were made to minimize suffering. One month within LSCD model establishment and cell transplantation, rabbits were monitored by slit lamp weekly under general anesthesia.

The right eye of each rabbit was used to establish the LSCD model by limbal resection, and the left eye served as control. After systemic and ocular surface anesthesia, rabbits underwent limbal lamellar keratectomy, 2 mm wide and 0.2 mm deep, and corneal epithelial removal. One month after the operation, the rabbit was identified as LSCD by cornea scores, which were composed of corneal neovascularization, opacity, and fluorescein staining. The scoring criterion was shown in S3 Table [26].

At 1 month after the formation of LSCD, 28 rabbits were randomly divided into three groups and received cell transplantation (12 rabbits in ESC-CEC group, 12 rabbits in LSC group, 4 rabbits in dAM group). A 360-degree conjunctival peritomy was made, and fibrovascular pannus presenting over the cornea was removed. The human amniotic membrane with cultivated ESC-CECs or LSCs or dAM alone was placed on the bare sclera and corneal stroma with the epithelial side oriented upward, and sutured with interrupted 10–0 nylon sutures. The conjunctiva was then sutured onto the limbus. Postoperatively, a contact lens was placed for 1 week, and 1% dexamethasone was given 4 times per day. After epithelialization was complete, 1% cyclosporine A eyedrops were administered 4 times per day. The ocular surface and cornea scoring was monitored weekly. At 1, 3, 7, 14, 30 days after the transplantation, rabbits were sacrificed by overdose sodium pentobarbital anesthesia to obtain the cornea tissue.

### Statistical analysis

All results were presented as Mean ± SEM and analyzed statistically by Stata 10 software. Analysis of variance was performed to compare the PCR result and the flow cytometry result. Paired t-test was used to compare the T lymphocyte proliferation assay and NK cytotoxicity assay. Differences were considered significant when p < 0.05. All results were repeated at least three times.

## Results

### Differentiation and identification of ESC-CECs

**Fig 1:**
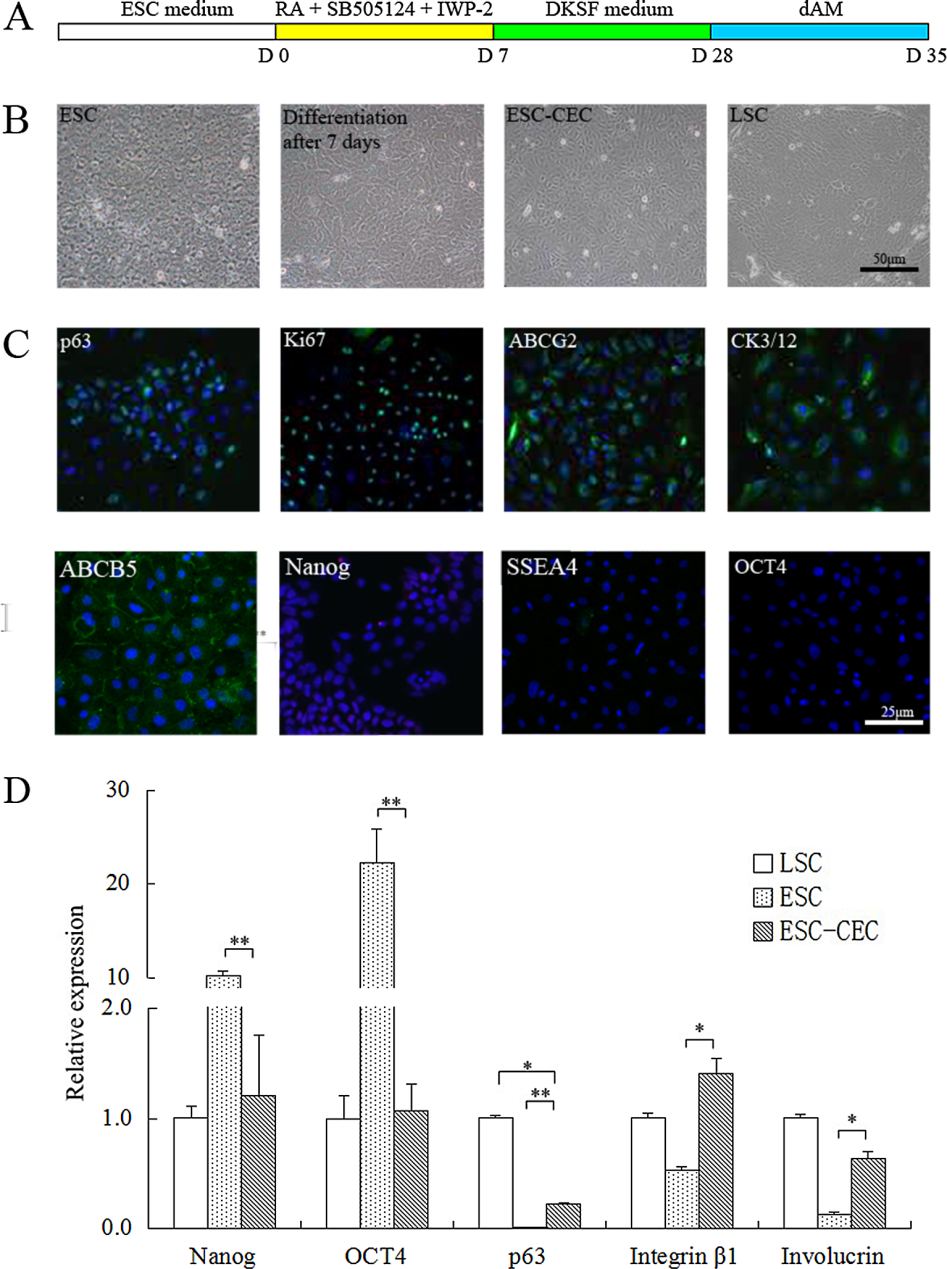
The character and identification of ESC-CECs in vitro. During the differentiation period of about 1 month (A), ESCs changed gradually into a epithelial morphology (B). The ESC-CECs were positive for LSC markers (green, C) and negative for pluripotent marker. The mRNA of LSC markers were high while that of pluripotent markers were low (n = 3, D). * p<0.05, ** p<0.01.

After 1 month of differentiation (Fig 1A), ESC-CECs were successfully obtained from human ESCs. During the differentiation, the cells changed gradually into an epithelial morphology (Fig 1B). Finally, the ESC-CEC cluster was composed of flatter cells and showed cobblestone appearance, which was similar with human LSCs (Fig 1B). The immunofluorescence staining revealed that the ESC-CECs were positive for stemness marker p63, Ki67 and ABCG2, and corneal epithelium marker CK3/12, especially they were positive for the new-found LSC marker ABCB5, but they were negative for pluripotent markers Nanog, SSEA4 and OCT4 (Fig 1C). The PCR showed that the ESC-CECs expressed a low level of pluripotent marker Nanog and OCT4, which was high in the ESCs. The expression of LSCs markers p63 and Integrin β1 was higher in the ESC-CECs than ESCs, the same to the terminal differentiation marker Involucrin (Fig 1D). These results indicated that ESC-CECs owned the character of LSCs and corneal epithelial cells but not ESCs.

### Transplantation of ESC-CECs in the LSCD model

**Fig 2:**
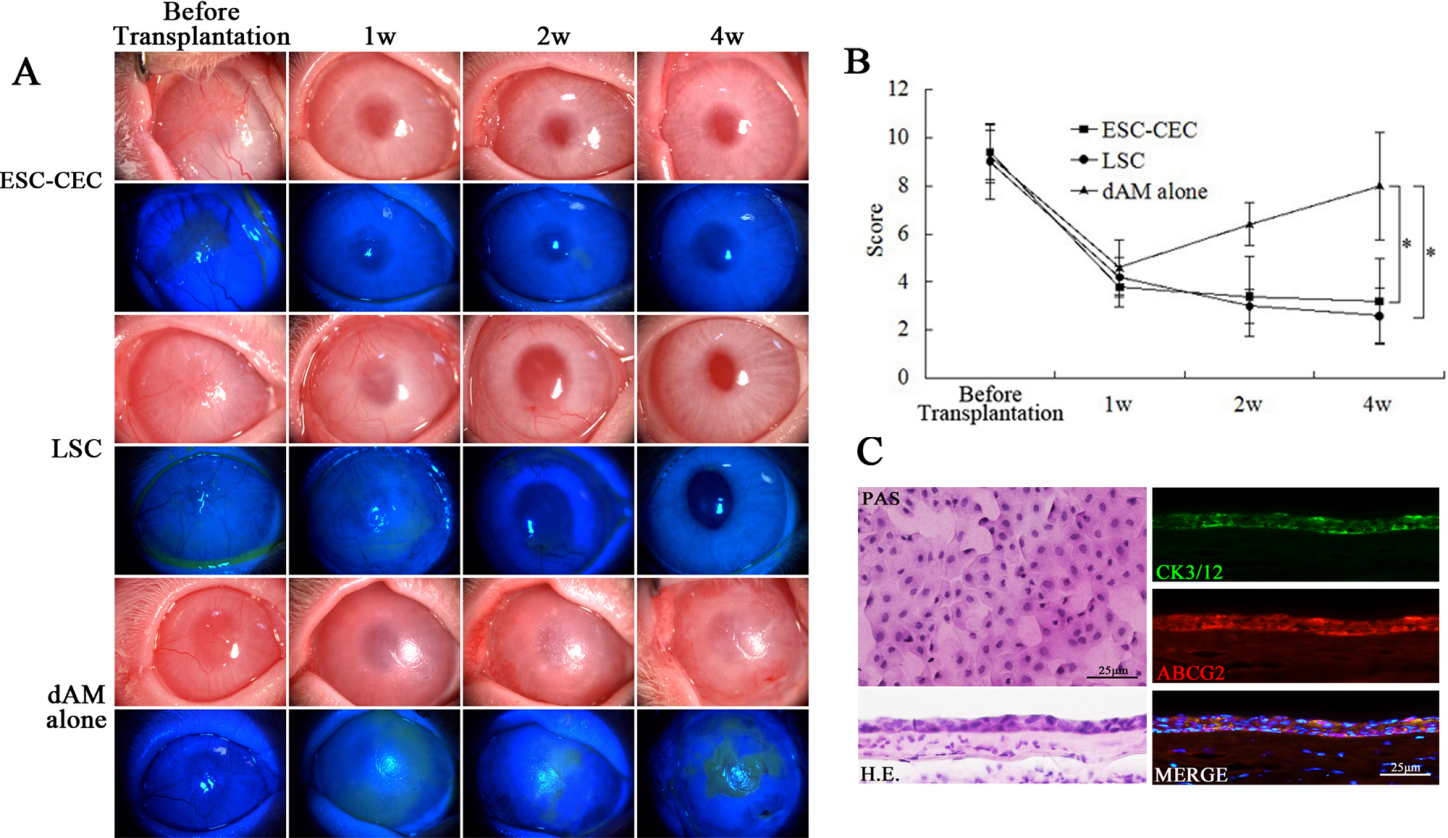
The clinical outcome of cell transplantation in rabbit with LSCD. Representative slit-lamp photograph and fluorescein staining revealed the ocular surface condition at different time after transplantation (A). The cornea score displayed the tendency after transplantation (n = 4, B). PAS staining, H.E. and immunofluorescence staining (C) showed a well epithelial structure. * p<0.05, ** p<0.01.

We further explored the biological function of ESC-CECs in the rabbit LSCD model. The result showed that the ESC-CECs could repair the conjunctivalized epithelium and restore corneal transparency. Before transplantation, the cornea was covered by the conjunctival epithelium and new blood vessels, and epithelial defect was illustrated by fluorescein staining (Fig 2A). After transplantation, the ocular surface condition in eyes receiving ESC-CECs and LSCs improved continuously. In both groups, the cornea became more and more transparent, without epithelial defect, and the new blood vessels disappeared gradually. At 1 month after transplantation, the ocular surface was still stable (Fig 2A). However, dAM alone transplantation did not exhibit a good therapeutic effect. Though there was no new blood vessel and little epithelial defect 1 week after transplantation, the new blood vessels and conjucntiva began to invade at 2 weeks, and the situation constantly worsened until 1month (Fig 2A). The cornea score displayed a same tendency. In all groups the score decreased significantly (approximately from 9 to 4) 1 week after transplantation, then the score remained stable in ESC-CECs and LSCs group but increased in dAM alone group (4 rabbits in each group, Fig 2B).

In the group with ESC-CEC transplantation, the PAS and H.E. stainings showed an intact epithelial structure on the cornea (Fig 2C). The epithelium was positive for corneal epithelial cell marker CK3/12 and limbal stem cell marker ABCG2 (Fig 2C). These results suggested that ESC-CECs could repair the epithelium, not structurally but functionally.

### Gene expression difference between ESC-CECs and LSCs

**Fig 3:**
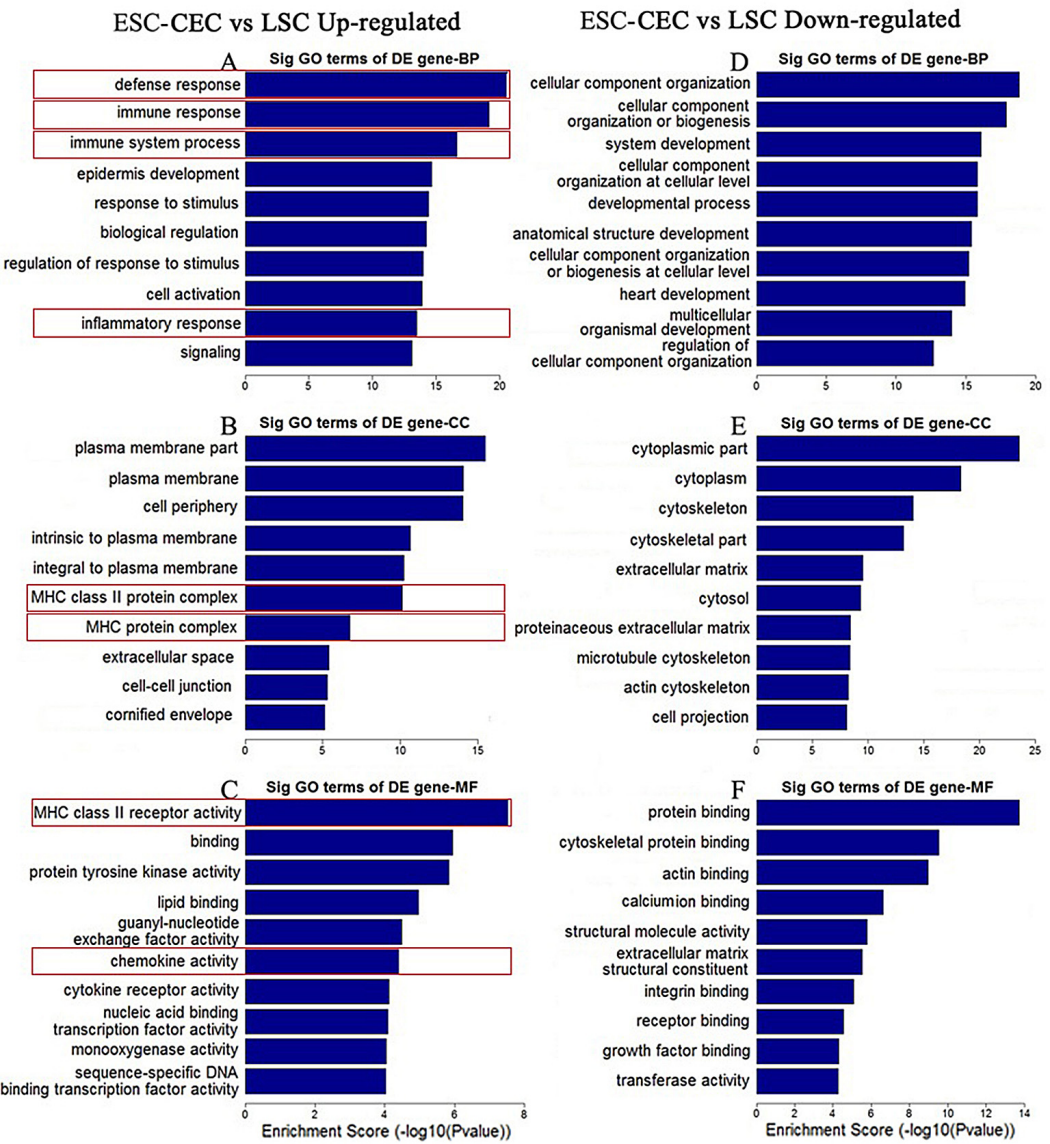
The top 10 GO terms significantly changed in ESC-CECs. The enrichment score quantified the change extend of down-regulated (A, B, C) or up-regulated (D, E, F) GO terms. Immune related GO terms were labeled by red frame.

To understand the immunological properties of ESC-CECs, we first got a profile of gene expression by genome microarray compared with LSCs. The GO analysis revealed that many GO terms significantly down-regulated in the ESC-CECs were related with the immune function. Of all down-regulated GO terms, defense response, immune response, immune system process, and inflammatory response ranked 1^st^, 2^nd^, 3^rd^, and 9^th^, respectively, in BP; MHC class II protein complex and MHC protein complex ranked 6^th^ and 7^th^, respectively, in CC; MHC class II receptor activity and chemokine activity ranked 1^st^ and 6^th^, respectively, in MF (Fig 3A, 3B and 3C). On the other hand, of the top ten up-regulated GO terms in BP, CC, and MF, none was related with immune function (Fig 3D, 3E and 3F). Furthermore, we analyzed the DE genes in these GO terms (Table 2). Many of them were of chemokine family and MHC class II protein, which meant that the ESC-CECs expressed less MHC-II and had a lowly chemotactic effect.

**Table 2:** Immune function related DE genes that greatly down regulated in ESC-CECs.

Genbank Accession	Gene Symbol	Description	Fold Change
NM_004847	AIF1	Homo sapiens allograft inflammatory factor 1 (AIF1), transcript variant 2	140
NM_013314	BLNK	Homo sapiens B-cell linker (BLNK), transcript variant 1	65
NM_002989	CCL21	Homo sapiens chemokine (C-C motif) ligand 21 (CCL21)	236
NM_002984	CCL4	Homo sapiens chemokine (C-C motif) ligand 4 (CCL4), transcript variant 1	82
NM_001174104	CD14	Homo sapiens CD14 molecule (CD14), transcript variant 3	65
NM_197947	CLEC7A	Homo sapiens C-type lectin domain family 7, member A (CLEC7A), transcript variant 1	58
NM_005211	CSF1R	Homo sapiens colony stimulating factor 1 receptor (CSF1R)	94
NM_001565	CXCL10	Homo sapiens chemokine (C-X-C motif) ligand 10 (CXCL10)	53
NM_002416	CXCL9	Homo sapiens chemokine (C-X-C motif) ligand 9 (CXCL9)	91
NM_001008540	CXCR4	Homo sapiens chemokine (C-X-C motif) receptor 4 (CXCR4), transcript variant 1	169
NM_021187	CYP4F11	Homo sapiens cytochrome P450, family 4, subfamily F, polypeptide 11 (CYP4F11), transcript variant 1	114
NM_005218	DEFB1	Homo sapiens defensin, beta 1 (DEFB1)	62
NM_004433	ELF3	Homo sapiens E74-like factor 3 (ets domain transcription factor, epithelial-specific) (ELF3), transcript variant 1	61
NM_005252	FOS	Homo sapiens FBJ murine osteosarcoma viral oncogene homolog (FOS)	1220
NM_004951	GPR183	Homo sapiens G protein-coupled receptor 183 (GPR183)	145
NM_002122	HLA-DQA1	Homo sapiens major histocompatibility complex, class II, DQ alpha 1 (HLA-DQA1)	385
NM_020056	HLA-DQA2	Homo sapiens major histocompatibility complex, class II, DQ alpha 2 (HLA-DQA2)	223
NM_001243962	HLA-DQB1	Homo sapiens major histocompatibility complex, class II, DQ beta 1 (HLA-DQB1), transcript variant 3	257
NM_001243962	HLA-DQB1	Homo sapiens major histocompatibility complex, class II, DQ beta 1 (HLA-DQB1), transcript variant 3	70
NM_019111	HLA-DRA	Homo sapiens major histocompatibility complex, class II, DR alpha (HLA-DRA)	870
NM_002124	HLA-DRB1	Homo sapiens major histocompatibility complex, class II, DR beta 1 (HLA-DRB1), transcript variant 1	78
NM_021983	HLA-DRB4	Homo sapiens major histocompatibility complex, class II, DR beta 4 (HLA-DRB4)	465
NM_002125	HLA-DRB5	Homo sapiens major histocompatibility complex, class II, DR beta 5 (HLA-DRB5)	223
NM_173843	IL1RN	Homo sapiens interleukin 1 receptor antagonist (IL1RN), transcript variant 4	239
NM_002163	IRF8	Homo sapiens interferon regulatory factor 8 (IRF8)	54
NM_002534	OAS1	Homo sapiens 2'-5'-oligoadenylate synthetase 1, 40/46kDa (OAS1), transcript variant 2	57
NM_000952	PTAFR	Homo sapiens platelet-activating factor receptor (PTAFR), transcript variant 3	79
NM_002922	RGS1	Homo sapiens regulator of G-protein signaling 1 (RGS1)	897
NM_003268	TLR5	Homo sapiens toll-like receptor 5 (TLR5)	64
NM_003820	TNFRSF14	Homo sapiens tumor necrosis factor receptor superfamily, member 14 (TNFRSF14)	121
NM_004624	VIPR1	Homo sapiens vasoactive intestinal peptide receptor 1 (VIPR1), transcript variant 1	56

### Expression of MHC and co-stimulatory molecules on ESC-CECs

**Fig 4:**
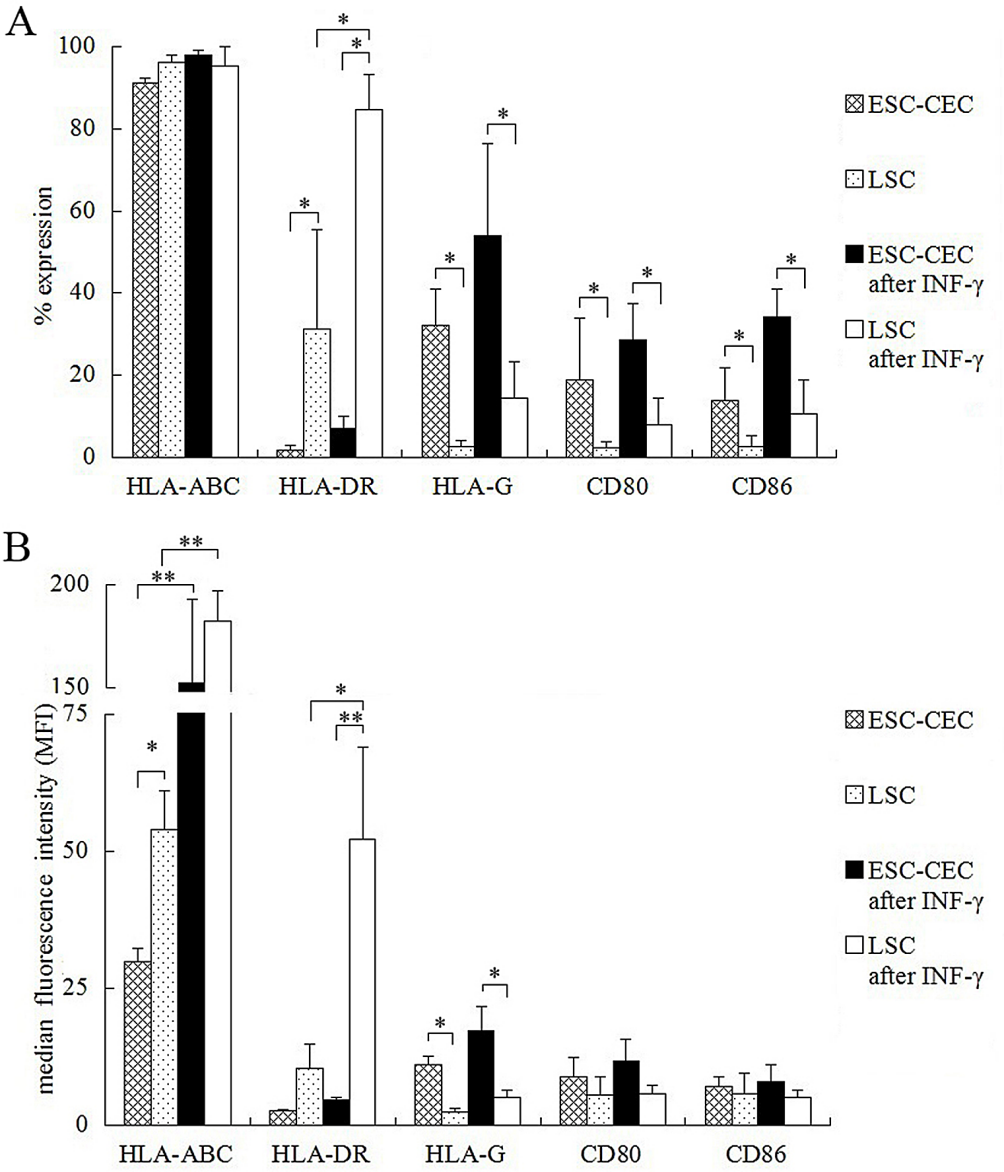
The flow cytometry of MHC and co-stimulatory molecules on ESC-CECs. The % expression (A) and MFI (B) was used for comparison of molecules on different cells and conditions. n = 3, * p<0.05, ** p<0.01.

According to genome microarray result, the expression of MHC and co-stimulatory molecules on ESC-CECs was investigated by flow cytometry. MHC class I molecule (MHC-I) was detected by HLA-ABC antibody, and MHC class II molecule (MHC-II) was detected by HLA-DR antibody. The % expression and median fluorescence intensity (MFI) were used for comparison. Almost all ESC-CECs and LSCs expressed HLA-ABC (p>0.05), but the MFI of ESC-CECs was weaker than that of LSCs (29.8 ± 2.5 vs 54.0 ± 7.1, p<0.05, Fig 4B). After INF-γ stimulation, both ESC-CECs and LSCs had an increased MFI (both p<0.01, Fig 4B), but the MFI between them showed no difference (p>0.05). Fewer ESC-CECs expressed HLA-DR than LSCs (1.9 ± 1.0 vs 31.4 ± 24.1, p<0.05, Fig 4A), though there was no difference in MFI (p>0.05). After INF-γ stimulation, the HLA-DR expression in ESC-CECs did not increased (both % expression and MFI p>0.05) while its expression in LSCs increased (% expression: 31.4 ± 24.1 vs 84.7 ± 8.4, p<0.05; MFI: 10.4 ± 4.3 vs 52.2 ± 16.8, p<0.05, Fig 4A and 4B). As result, the MFI of ESC-CECs was much weaker than that of LSCs (4.6 ± 0.4 vs 52.2 ± 16.8, p<0.01, Fig 4B). Compared with LSCs, ESC-CECs expressed greater HLA-G (% expression: 32.2 ± 8.8 vs 2.8 ± 1.4, p<0.05; MFI: 11.0 ± 1.7 vs 2.5 ± 0.6, p<0.05, Fig 4A and 4B). The INF-γ stimulation did not change the expression in ESC-CECs and LSCs (both % expression and MFI, p>0.05). The co-stimulatory molecules CD80 and CD86 shared a same expression character. More ESC-CECs were positive for CD80 and CD86 than LSCs (CD80: 19.0 ± 15.0 vs 2.2 ± 1.5, p<0.05; CD86: 13.9 ± 7.9 vs 2.6 ± 2.6, p<0.05, Fig 4A). The INF-γ stimulation did not change the expressions of CD80 and CD86 in ESC-CECs and LSCs (all p>0.05). S1 Table and S2 Table listed the detailed information about the expression percentage and MEI of MHC and co-stimulatory molecules on ESC-CECs and LSCs.

### T cell proliferation stimulated by ESC-CECs

**Fig 5:**
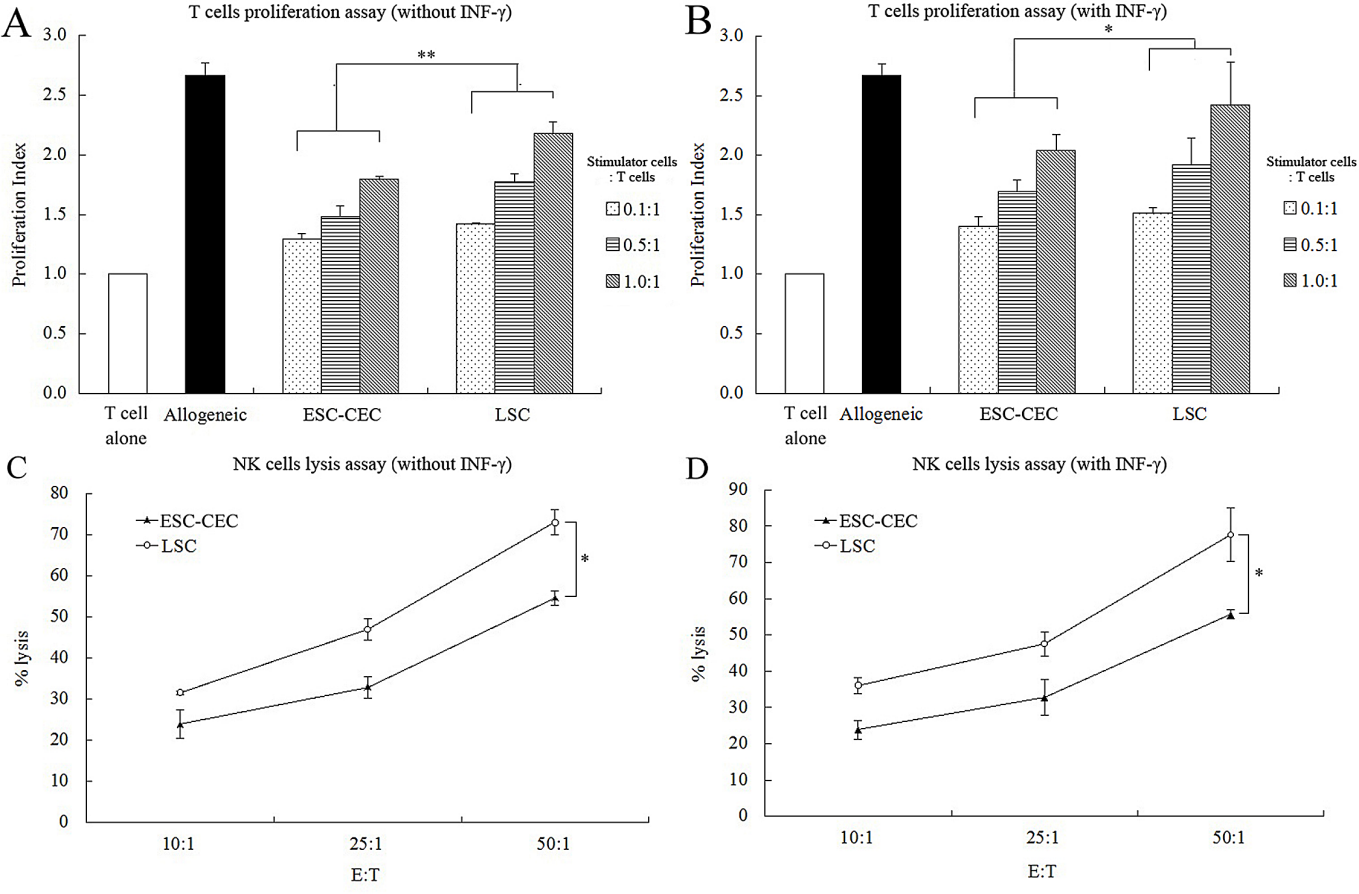
The T cells proliferation assay and NK cells lysis assay. ESC-CECs stimulated a weaker T cells proliferation (A) and were less susceptible to NK cells lysis (C) compared with LSCs. The situation didn’t change after INF-γ treatment (B, D). n = 4, * p<0.05, ** p<0.01.

T lymphocyte proliferation assay was performed to evaluate the ESC-CECs immunogenicity in vitro. The result showed that the proliferation index of both ESC-CECs and LSCs was lower than that of allogeneic T cells (Fig 5A). At any ratio of ESC-CECs or LSCs to T cells, the proliferation index of ESC-CECs was lower than that of LSCs (p < 0.01, Fig 5A). After stimulated by INF-γ, the proliferation index of ESC-CECs was also lower than that of LSCs at any ratio (p < 0.05, Fig 5B). It was remarkable that the proliferation index of ESC-CECs did not increase after INF-γ stimulation (p > 0.05), while the proliferation index of LSCs increased (p < 0.05, Fig 5A and 5B). All these results indicated a low immunogenicity of ESC-CECs in vitro.

### NK cytotoxicity to ESC-CECs in vitro

NK cytotoxicity assay was performed to evaluate the ESC-CEC susceptibility to NK cells attack. The result showed that the % lysis of both ESC-CECs and LSCs increased as the E:T ratio increased. Fewer ESC-CECs were killed by NK cells than LSCs at any E:T ratio (p < 0.01, Fig 5C). After INF-γ stimulation, the % lysis of both ESC-CECs and LSCs did not change (both p > 0.05, Fig 5C and 5D), and the ESC-CECs were also less susceptible to NK cells attack than LSCs (p < 0.01, Fig 5D). These results suggested ESC-CECs had a low susceptibility to NK cells attack.

### Immune reaction after ESC-CEC transplantation

**Fig 6:**
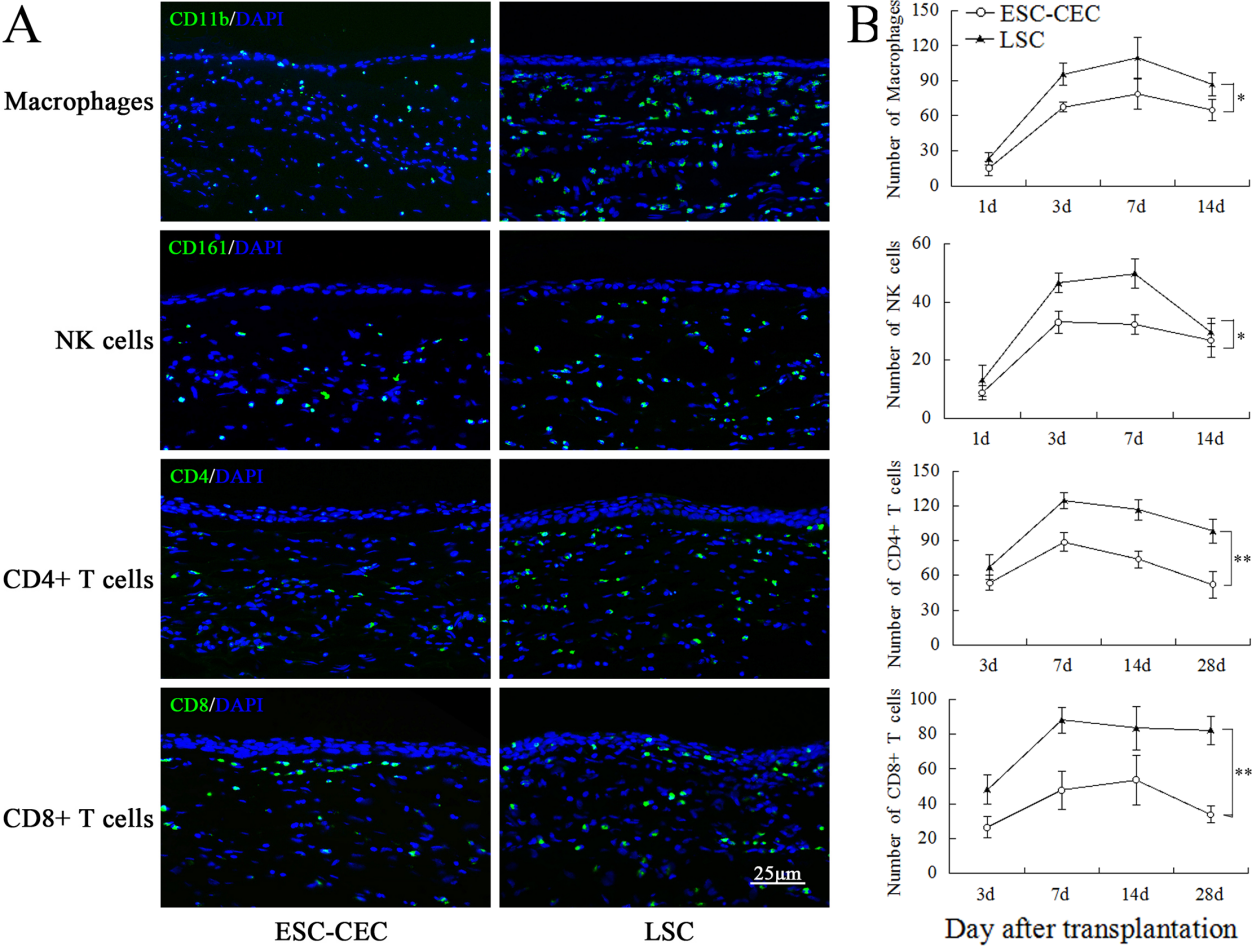
The immune cells infiltration after ESC-CEC and LSC transplantation. Representative immunofluorescence pictures of macrophages and NK cells infiltration 3 days after transplantation, CD4+ and CD8+ T cells infiltration 7 days after transplantation (green A). All immune cells were fewer in the ESC-CEC transplantation group (n = 4, B). * p<0.05, ** p<0.01.

We analyzed the character of immune reaction after ESC-CEC transplantation compared with LSC transplantation. The number and distribution of macrophages, NK cells, CD4+ T cells, CD8+ T cells were labeled by CD11b, CD161, CD4, and CD8 antibody, respectively. Macrophages and NK cells migrated into the cornea at 1 d after transplantation and constantly increased until 3~7 d. Then they decreased gently. Both groups shared the same trend, but the number of macrophages and NK cells in the ESC-CEC transplantation group was fewer than that in the LSC transplantation group (Fig 6A). Both CD4+ and CD8+ T cells appeared at 3 d after transplantation and peaked in number at about 7~14 d. Fewer CD4+ and CD8+ T cells migrated into the cornea in the ESC-CEC group compared with the LSC group (Fig 6A). It was worth noting that there were also a small amount of macrophages and T cells, but not NK cells, in the cornea with LSCD before cell transplantation while normal cornea was negative (data was not shown). For each stained cornea section,we chose the central cornea (400μm wide) and peripheral cornea (3mm from the center, 200μm wide on both sides) to count positive immune cells for analysis. The result illustrated a same trend, all immune cells were fewer in ESC-CEC group (Fig 6B). The less immune cells infiltration denoted a weaker rejection after ESC-CEC transplantation.

## Discussion

Cell transplantation remains the major approach to the treatment of LSCD, and the biological properties of donor cells are a key factor for clinical efficacy [27]. In the present study, the corneal epithelial-like cells derived from ESCs were not only sufficient but also proved to fix the damaged corneal epithelium effectively. More importantly, the ESC-CECs exhibited a low immunogenicity in various aspects.

For bilateral LSCD, transplantation of allogeneic LSCs or autologous oral mucosal epithelial cells is the only choice. But the immune rejection after allogeneic LSCs limits the rate of success, and autologous oral mucosal epithelial cells have a weak anti-angiogenesis function [28, 29]. Inducing pluripotent stem cells into corneal epithelial-like cells provides another choice, and great advances have been achieved recently. Our group successfully obtained corneal epithelial-like cells from human ESCs by RA, SB505124, IWP-2 induction system. The ESC-CECs expressed not only markers of LSCs but also stemness markers, and the ocular surface of rabbits with LSCD improved greatly after ESC-CEC transplantation. These results were consistent with previous studies [30, 31]. Also, it should be noticed that different cell lines and culture medium sometimes displayed various differentiation tendencies [32]. At present study, we mainly focus on the differentiation and optimization of H1 line. To fully demonstrate the efficacy of the induction system, other human ESC line, even induced pluripotent stem cells will be used in the future experiment.

MHC molecules are the main antigen in the rejection of allograft transplantation, and in humans they are known as HLA. MHC-I is expressed on all cells with nucleus, while MHC-II is mainly expressed on B lymphocytes, macrophages, and dendritic cells. In theory, the level of MHC-I and MHC-II together determines the cell immunogenicity and the rejection after transplantation [33]. In this study, the ESC-CECs expressed less MHC-I and MHC-II than the LSCs. It was in line with the fact that ESCs and their derivatives were at a low level in MHC molecules [16, 17].

HLA-G is a non-classical HLA class I molecule found in 1987 and high expressed in placental trophoblast cells for maternal-fetal immune tolerance [34]. Later, it emerged as an important immunomodulatory molecule, because it caused a direct inhibitory effect on proliferation and cell killing function of NK cells and cytotoxic T cells, prevented maturation and function of dendritic cells, and reduced proliferative responses of allogeneic CD4+ T cells [35–37]. Moreover, it was found to be expressed by the corneal epithelial, stromal, and endothelial cells, and contribute to the immune privilege of the eye [38, 39]. The ESC-CECs expressed more HLA-G compared with LSCs, which was beyond our expectation. Although its exact mechanism and detailed function in ESC-CECs need further investigations, we suppose that the immunomodulatory effect of HLA-G partially contributes to the low immunogenicity of ESC-CECs.

Our finding that some ESC-CECs expressed co-stimulatory molecules CD80 and CD86 needs to be further discussed, because the absence of MHC-II and co-stimulatory molecules is usually a sign of low immunogenicity [40]. Co-stimulatory molecules are expressed on antigen presenting cells and provide the second signal when T cells are activated. However, the ESC-CECs are not antigen presenting cells, and the expression of co-stimulatory molecules may be meaningless. Moreover, without MHC-II expression, only co-stimulatory molecules in the ESC-CECs can not provide a way of direct antigen presenting, which was proved by the result that the ESC-CECs provoked a weak T cell proliferation. Therefore, the expressions of CD80 and CD86 are more likely to be a side effect of differentiation, and can not be seen as a sign of enhanced immunogenicity.

A low level of MHC molecules theoretically increases the susceptibility to NK cells attack as it is an important way to defense pathogenic microorganisms in normal condition [41]. To assess the situation of ESC-CECs, NK cytotoxicity assay was performed in our study. The result revealed that the ESC-CECs were less possible to be lysed by NK cells than LSCs, and they were not impacted by INF-γ. After transplantation in vivo, there were fewer NK cells migrating into the cornea. Previous studies also suggested a conflicting result of substantial and negligible in vitro NK responses against murine ESCs, presumably depending on the experimental conditions [42, 43]. As for human ESCs, human NK cells could not recognize ESCs effectively in vitro only after additional NK stimulation [44]. So the low level of MHC in ESC-CECs is not a critical matter for NK cells attack.

Although the ocular surface is considered as an immune privileged organ, the experience of ex vivo expanded allogeneic LSC transplantation tells us that there are varying degrees of inflammatory responses in the early stage and continuous chronic rejection in the late period despite immunosuppressive agents [24, 45]. The inflammatory microenvironment can help up-regulate MHC molecule expression, increase the recognition by leukocytes, promote the infiltration of activated immune cells, and change the immunomodulatory cell function, and thus affects the immunogenicity, function, and survival of donor cells [46, 47]. After in vitro treatment of INF-γ, which is a main proinflammatory cytokine, the expression of most MHC molecules and co-stimulatory molecules in ESC-CECs did not change, and the result of T cell proliferation and NK cell lysis to ESC-CECs was not affected. The only change was the MFI of MHC-I expression. Moreover, in vivo ESC-CECs indeed led to a weak immune reaction indicated by less immune cells infiltration. So the inflammation exhibited little influence on the immunogenicity of ESC-CECs.

The in vivo immunogenicity of ESC-CECs was evaluated in rabbits with LSCD, which means a xenogeneic transplantation. Some differences exist in the immune response after xenogeneic and allogeneic transplantation. For example, the major antigen in a xenograft is not limited to MHC molecules. The hyperacute rejection in xenogeneic transplantation is severer. The antigen presence of a xenograft occurs almost by an indirect pathway [48, 49]. Despite this, we compared ESC-CECs with LSCs in the same background to overcome the xenogeneic transplantation problem, and ESC-CECs showed a weaker immune response than LSCs. Further studies, such as assessing the ESC-CEC immunogenicity in mice with humanized immune system, are needed to evaluate the allogeneic immunogenicity of ESC-CECs.

## Conclusions

The corneal epithelial-like cells derived from ESCs present a similar character and function with LSCs. More importantly, the ESC- CECs, seem to be promising in the clinical reconstruction of ocular surface for their low immunogenicity both in vitro and in vivo.

## Supporting Information

S1 Fig The Representative flow cytometry analysis pictures. (TIF)

S1 Table Expression percentage of MHC and co-stimulatory molecules on ESC-CECs and LSCs. (DOC)

S2 Table MFI of MHC and co-stimulatory molecules on ESC-CECs and LSCs. (DOC)

S3 Table The cornea scoring criterion. (DOC)
