# Supplementary figures and images for: Immunological Properties of Corneal Epithelial-Like Cells Derived from Human Embryonic Stem Cells
Source: PLoS One. 2016 Mar 15;11(3):e0150731. doi: 10.1371/journal.pone.0150731 (PMC4792422; doi:10.1371/journal.pone.0150731)

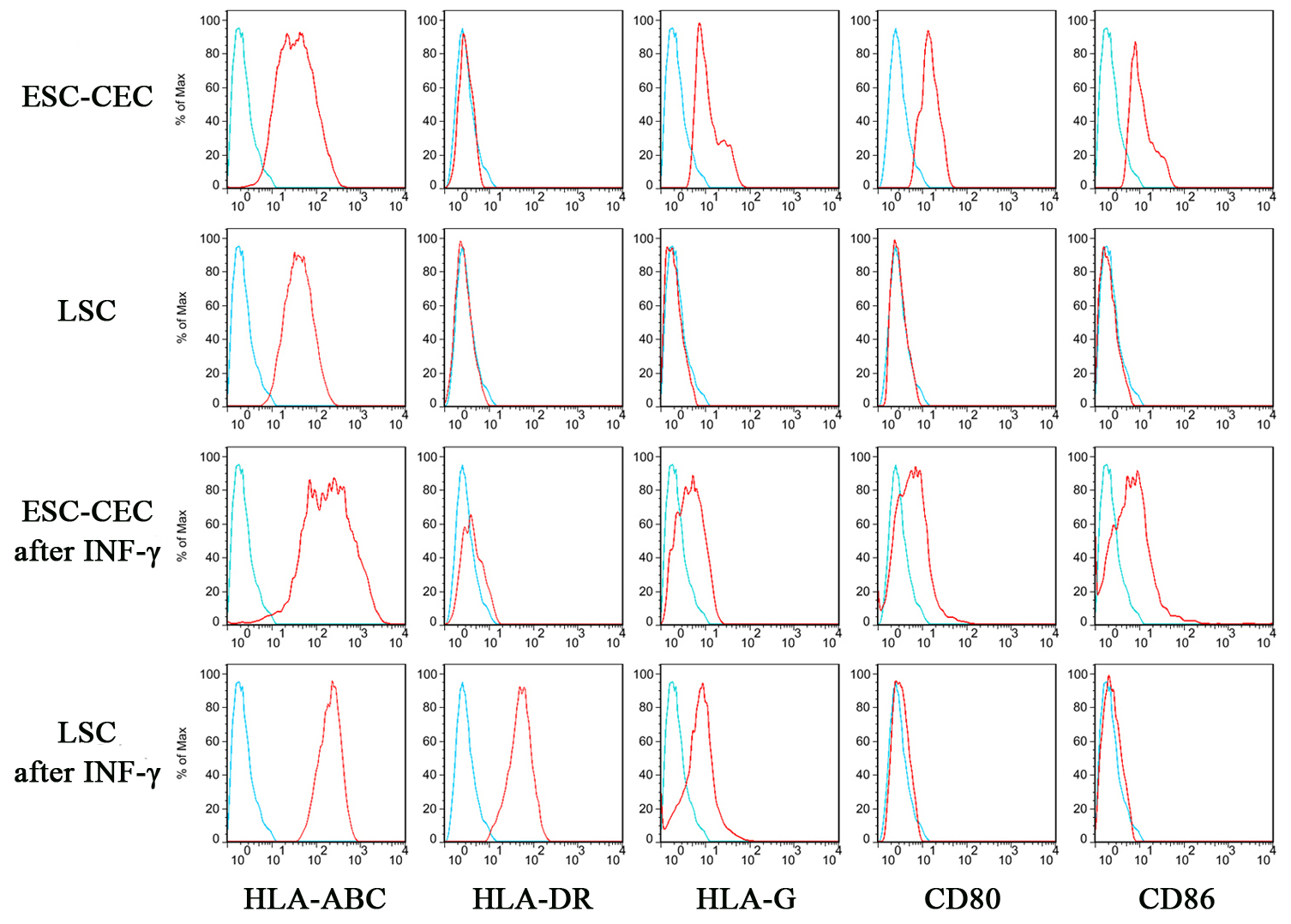

Supplement: S1 Fig — (TIF) [file pone.0150731.s001.tif]
