# Supplementary material for: Immunological Properties of Corneal Epithelial-Like Cells Derived from Human Embryonic Stem Cells
Source: PLoS One. 2016 Mar 15;11(3):e0150731. doi: 10.1371/journal.pone.0150731 (PMC4792422; doi:10.1371/journal.pone.0150731)
Supplement: S1 Table — (DOC) [file pone.0150731.s002.doc]

**Table S1.** **Expression percentage of MHC and co-stimulatory molecules on ESC-CECs and LSCs**

| % expression | HLA-ABC | HLA-DR | HLA-G | CD80 | CD86 |
| --- | --- | --- | --- | --- | --- |
| CEC | 91.2 ± 1.3 | 1.9 ± 1.0^*^ | 32.2 ± 8.8^*^ | 19.0 ± 15.0^*^ | 13.9 ± 7.9^*^ |
| LSC | 96.2 ± 1.7 | 31.4 ± 24.1^*^ | 2.8 ± 1.4^*^ | 2.2 ± 1.5^*^ | 2.6 ± 2.6^*^ |
| CEC-INF | 97.8 ± 1.4 | 7.1 ± 3.0^#^ | 54.0 ± 22.3^#^ | 28.6 ± 8.9^#^ | 34.2 ± 6.7^#^ |
| LSC-INF | 95.2 ± 4.8 | 84.7 ± 8.4^#^ | 14.6 ± 8.8^#^ | 7.9 ± 6.6^#^ | 10.7 ± 8.1^#^ |

^*,#^ p<0.05,
