# Supplementary material for: Immunological Properties of Corneal Epithelial-Like Cells Derived from Human Embryonic Stem Cells
Source: PLoS One. 2016 Mar 15;11(3):e0150731. doi: 10.1371/journal.pone.0150731 (PMC4792422; doi:10.1371/journal.pone.0150731)
Supplement: S2 Table — (DOC) [file pone.0150731.s003.doc]

**Table S2.** **MFI of MHC and co-stimulatory molecules on ESC-CECs and LSCs**

| median fluorescence  intensity (MFI) | HLA-ABC | HLA-DR | HLA-G | CD80 | CD86 |
| --- | --- | --- | --- | --- | --- |
| CEC | 29.8 ± 2.5^**^ | 2.6 ± 0.2 | 11.0 ± 1.7^*^ | 10.0 ± 3.6 | 7.1 ± 1.8 |
| LSC | 54.0 ± 7.1^##^ | 10.4 ± 4.3 | 2.5 ± 0.6^*^ | 2.3 ± 0.3 | 2.5 ± 0.4 |
| CEC-INF | 282.0 ± 81.5^**^ | 4.6 ± 0.4^**^ | 17.2 ± 4.4^#^ | 13.2 ± 3.9 | 14.6 ± 5.1 |
| LSC-INF | 146.5 ± 45.1^##^ | 52.2 ± 16.8^**^ | 5.0 ± 1.5^#^ | 4.7 ± 0.8 | 5.1 ± 1.4 |

^*,#^ p<0.05, ^**, ##^ p<0.01
