# Supplementary material for: Immunological Properties of Corneal Epithelial-Like Cells Derived from Human Embryonic Stem Cells
Source: PLoS One. 2016 Mar 15;11(3):e0150731. doi: 10.1371/journal.pone.0150731 (PMC4792422; doi:10.1371/journal.pone.0150731)
Supplement: S3 Table — (DOC) [file pone.0150731.s004.doc]

**Table S3.** **The cornea scoring criterion**

| items | 0 | 1 | 2 | 3 | 4 |
| --- | --- | --- | --- | --- | --- |
| opacity | No opacity | Slight opacity, veins of iris can be seen | Moderate opacity, veins of iris are not clear | Severe opacity, only pupil can be seen | Extremely severe opacity, pupil can’t be seen |
| fluorescein staining | No fluorescein staining | The area of fluorescein staining ≤ 1/4 quadrant | The area of fluorescein staining > 1/4 quadrant but ≤ 1/2 quadrant | The area of fluorescein staining > 1/2 quadrant but ≤ 3/4 quadrant | The area of fluorescein staining > 3/4 quadrant |
| neovascularization | No new vessels | The new vessels are within 2mm from limbus | The new vessels are on peripheral cornea and ≤ 1/2 quadrant | The new vessels are on peripheral cornea and >1/2 quadrant | The new vessels are on the whole cornea |
